# Functional network connectivity during Jazz improvisation

**DOI:** 10.1038/s41598-021-98332-x

**Published:** 2021-09-24

**Authors:** Victor M. Vergara, Martin Norgaard, Robyn Miller, Roger E. Beaty, Kiran Dhakal, Mukesh Dhamala, Vince D. Calhoun

**Affiliations:** 1 Tri-Institutional Center for Translational Research in Neuroimaging and Data Science (TReNDS), Georgia State University, Georgia Institute of Technology, and Emory University, 55 Park Place, Atlanta, GA 30303 USA; 2 School of Music, Georgia State University, Atlanta, GA USA; 3 Department of Psychology, Pennsylvania State University, University Park, PA USA; 4 Department of Radiology, Massachusetts General Hospital, Harvard Medical School, Athinoula A. Martinos Center for Biomedical Imaging, Charlestown, MA USA; 5 Department of Physics and Astronomy, Neuroscience Institute, Georgia State University, Atlanta, GA USA

**Keywords:** Cognitive neuroscience, Computational neuroscience, Emotion, Learning and memory, Motivation

## Abstract

One of the most complex forms of creativity is musical improvisation where new music is produced in real time. Brain behavior during music production has several dimensions depending on the conditions of the performance. The expression of creativity is suspected to be different whether novel ideas must be externalized using a musical instrument or can be imagined internally. This study explores whole brain functional network connectivity from fMRI data during jazz music improvisation compared against a baseline of prelearned score performance. Given that creativity might be affected by external execution, another dimension where musicians imagine or vocalize the music was also tested. We found improvisation was associated with a state of weak connectivity necessary for attenuated executive control network recruitment associated with a feeling of “flow” allowing unhindered musical creation. In addition, elicited connectivity for sensorimotor and executive control networks is not different whether musicians imagine or externalize (through vocalization) musical performance.

## Introduction

Creativity is an essential human trait supporting evolution through the ages up through inventions of our time. Typically, creativity has been studied using paradigms in which a process of generation results in the production of a creative product^1^. Recently, there has been increased interest in creative processes that occur in real time where idea generation and evaluation may occur in rapid succession or even simultaneously. One model behavior that has been studied extensively using neuroscience methods is musical improvisation especially within the style of jazz, for a review see^2^. Expert jazz musicians create novel musical output within given general constraints of style, specific constraints dictated by the current melody and harmony, and motoric constraints dictated by the instrument or voice apparatus^3^. In contrast to paradigms in which the creative product is not generated in real time (e.g. product invention or musical composition), improvisers have no opportunity to revise once the output is produced. Music improvisation thus offers a model to study spontaneous creativity under considerable temporal and motoric constraints.

One influential theoretical framework suggests improvisation is only possible because improvisers reuse learned patterns of notes inserted in the ongoing improvisation^3^. This view is supported by evidence of a high degree of repeated musical patterns in extant improvisations by experts^4,5^. These patterns are part of expert performers’ knowledgebase which also includes strategies for concatenating the known patterns, rules for generating new patterns, how the patterns relate to underlying harmonic and rhythmic contexts, which motor movements are needed to execute the patterns, and more general information about style and performance context^3^.

It has recently been suggested that creativity (including improvisation) requires a continuous process of idea generation/retrieval and evaluation and that this process aligns with specific brain network functionality^6^. The generation and evaluation of ideas is thought to be supported by an interplay between two major brain networks, the default mode network (DMN) and the executive control network (ECN). The DMN comprises a set of brain regions, including ventromedial prefrontal cortex (vmPFC) and posterior cingulate cortex (PCC), involved in generation of self-referential thought^7^. The vmPFC has a direct link with emotion^8^ while playing an important role in memory generalization^9^ and integration^10^. The PCC activates during freely unconstrained cognitive processes^11^ and when creating and consolidating coherent memory representations of complex events^12^. A previous study suggests that ideas may be generated or retrieved through internal consolidation of abstract representations in a bottom-up process supported by the DMN, which has also been implicated in mind wandering and other activities in which ideas are generated without constraints related to task demands^13^. With respect to improvisation, generated ideas may then be evaluated according to the current context and inserted into the ongoing improvisation through a process involving the ECN, which consists of the dorsolateral prefrontal (dlPFC) and dorsal parietal cortices^14^. Dorsal parietal and frontal cortices facilitate the preparation and execution of goal-directed stimuli and responses^15^. The ECN incorporates exogenous sensorial and more internal information to coordinate behavior^16^. Interaction of the ECN and DMN may therefore reflect a coordination of controlled and spontaneous processes during improvisation. In a simplified explanation, novel self-generated information flows from the DMN to find a concrete physical expression via ECN intervention. Indeed, activation in some areas of the ECN during musical improvisation, specifically the dlPFC, can be modulated by the improvisational context with higher constraints resulting in increased activation^17^.

The first study to implicate DMN in improvisation used a functional Magnetic Resonance Imaging (fMRI) paradigm where expert jazz pianists switched between playing prelearned and improvised material^18^. Compared to playing learned sequences, improvisation showed increased activation of DMN regions and concurrent deactivation of ECN regions. ECN deactivation was interpreted as attenuated evaluation, as experts typically insert ideas that fit the current context with minimal cognitive control necessary^19,20^. They referred to this deactivated state as hypofrontality which has previously been related to the state of flow^21^. Flow is specifically associated with high engagement in an interesting task to the point where brain activation related to self-referential processing is attenuated^22,23^. The results were duplicated in a different paradigm in which rhythmic language improvisation again elicited dissociated activity in medial and dorsolateral prefrontal cortices^24^. Interestingly, a later follow-up study in which expert participants engaged in interaction with another player saw increased activation in control areas associated with language production apparently interrupting the state of flow^25^.

During improvisation, the process of idea generation and evaluation occurs concurrently with other energy-demanding processes of perception and action. Brain interactions can occur in two ways. At one end, endogenous and exogenous processes can compete with each other, resulting in detrimental brain performance. This clash decreases performance when a failure to deactivate DMN creates interference for exogenous information processing during goal-directed task execution^26,27^. A recent model by Psyche Loui^28^ outlines the importance of the perception and action loop within the context of musical performance and improvisation. Any movement that triggers a sound will involve the temporal lobe in which the auditory signal is initially analyzed. From there, a dorsal network—consisting of the superior temporal gyrus, connected by the arcuate fasciculus to the frontal lobe—supports sensory-motor integration. At the same time, information about sound-object categories is sent via a ventral network from the middle temporal gyrus to inferior frontal regions through the uncinate and inferior longitudinal fascicule. While these processes are common to all music performance^29^, these networks may additionally underpin idea generation and evaluation during improvised music^28^. Indeed, musicians show higher fractional anisotropy (FA) in the arcuate fasciculus than non-musicians^30^. In addition, jazz musicians compared to non-improvising musicians showed higher tract volume and FA between mesial regions and left superior temporal gyrus and between mesial regions and right inferior frontal gyrus^31^. Differences between improvising and non-improvising musicians can also be seen in resting state functional connectivity where improvising musicians show higher integration of DMN and ECN^32^.

Previous literature has investigated anatomical and functional changes in connectivity, revealing definite neuroplasticity related to musical performance. To date, most functional connectivity studies have focused on a few regions of interests known to be involved in musical creativity^32,33^, leaving questions about whole-brain dynamics during improvisation. Moreover, most work has examined improvisation in the context of keyboard performance, with little attention paid to spontaneous creativity in other domains (e.g., vocal). Examining whole-brain dynamics during improvisation, beyond a single musical instrument, can provide further insight into the complex and multi-modal nature of spontaneous human creativity.

In a recent study, we examined functional activity and connectivity with expert jazz improvisers who either vocally improvised or recalled prelearned melodies^33^. We chose a vocalization task as jazz musicians often practice vocally independent of their instrumental specialization^34^. Critically, our prior study was limited to traditional activation contrasts and ROI-based connectivity analysis, and we did not examine whole-brain dynamics. In line with the theory of hypofrontality, the results showed decreased connectivity between left frontal, lateral premotor cortex, supplementary motor area and cerebellum during improvisation. Importantly, the context of the improvisation task was very well known to the participants (improvising on the common 12-bar blues) with the goal of minimizing external constraints and promoting a flow state. Interestingly, we also found decreased connectivity between regions of interest during improvisation in a study where we collected electroencephalography (EEG) while participants played or imagined prelearned or improvised short isochronous melodies^35^.

The current work substantially extends our previous study of expert jazz musicians by examining whole-brain functional connectivity during vocal and imagined improvisation. We comprehensively investigate whole-brain functional network connectivity while musicians either imagine or perform novel or prelearned vocal melodies. This data-driven analysis allowed us to examine how the whole brain dynamically functions during spontaneous musical creativity, providing a significant expansion of prior work focusing on localized activity and regions of interest^25^. Our previous studies contrasting prelearned and improvisation tasks would indicate that improvisation involves less connectivity between regions. Therefore, we hypothesized that whole brain connectivity in the current analysis would also be attenuated during improvisation.

## Methods

### Subjects

A total of twenty-one male advanced jazz improvisers (4 left-handed, 17 right-handed; mean age ± standard deviation [SD] = 30.67 ± 9.97 years) were included in this study. Participants had at least 6 years of professional experience (mean ± SD = 20.48 ± 11.1 years) on jazz improvisation, see participants description in^33^. All participants had previous education in a University System School of Music; average schooling years for all participants was 16.21 ± 1.86 years. Reading music scores was also required for all participants. Primary instruments included piano (n = 5), saxophone (n = 10), guitar (n = 1), trumpet (n = 2), drums (n = 1), trombone (n = 1), and bass (n = 1). All participants had normal or corrected to normal vision and reported normal neurological history. Participants provided written and signed informed consent forms and were compensated for their participation in the experiment. The Institutional Review Board for Joint Georgia State University and Georgia Institute of Technology Center for Advanced Brain Imaging, Atlanta, Georgia, approved this study. All methods were performed in accordance with the relevant guidelines and regulations.

### fMRI scanning protocol

All fMRI images were acquired on a 3-Tesla Siemens scanner available at Georgia State University and Georgia Institute of Technology Center for Advanced Brain Imaging, Atlanta, Georgia. Acquisition protocol was done with T2* weighted gradient echo-planar imaging sequence: echo time (TE) = 30 ms, repetition time (TR) = 1970 ms, flip-angle = 90°, field of view = 204 mm, matrix size = 68·68, voxel size = 3·3·3 mm^3^, and 37 interleaved axial slices with a thickness of 3 mm each. High-resolution anatomical images were acquired for anatomical references using a magnetization-prepared rapid gradient-echo sequence with TR = 2250 ms, TE = 4.18 ms, flip-angle = 9°, and voxelsize = 1·1·1 mm^3^.

Data were preprocessed by using Statistical Parametric Mapping (SPM12; www.fil.ion.ucl.ac.uk/spm). The preprocessing steps involved slice timing correction, motion correction, coregistration to individual anatomical image, and normalization to Montreal Neurological Institute (MNI) template^36^. Spatial smoothing of normalized image was done with an 8-mm isotropic Gaussian kernel.

Data from all subjects were subject to a “cleaning” group independent component analysis (gICA) using the GIFT software (https://trendscenter.org/software/gift/) to obtain a set of independent components. Artifactual components were detected and discarded based on their relationship with white matter, cerebrospinal fluid and frequency. Data was reconstructed and a second gICA was performed resulting in 100 components. We employed the package ICASSO currently integrated in GIFT to ensure a good quality of gICA decomposition^37^.

We selected a subset of 39 non-artifactual components representing the brain resting state networks (RSNs) of interest. RSNs were organized in functional domains: Basal Ganglia (BG), Auditory (AUD), Sensorimotor (SEN), Cerebellum (CER), Primary Visual (PrimVIS), High Visual (HighVIS), ventral default mode network (vDMN), dorsal default mode network (dDMN), salience (SAL), executive control network (ECN) and language (LAN). As sanity check, we confirmed RSN—domain matching by examining spatial overlap with Stanford resting state masks^38^. RSN time courses were detrended up to third-order trends and filtered with a Butterworth fifth-order filter of bandwidth [0.01, 0.15] Hz. Static functional network connectivity (FNC) was assessed using Pearson correlation between gICA component time courses within each task. Pearson Z transformation was applied to every pair of time courses for a total of 741 (39*38/2) connectivity values per subject and task.

### Condition and task paradigms

**Figure 1:**
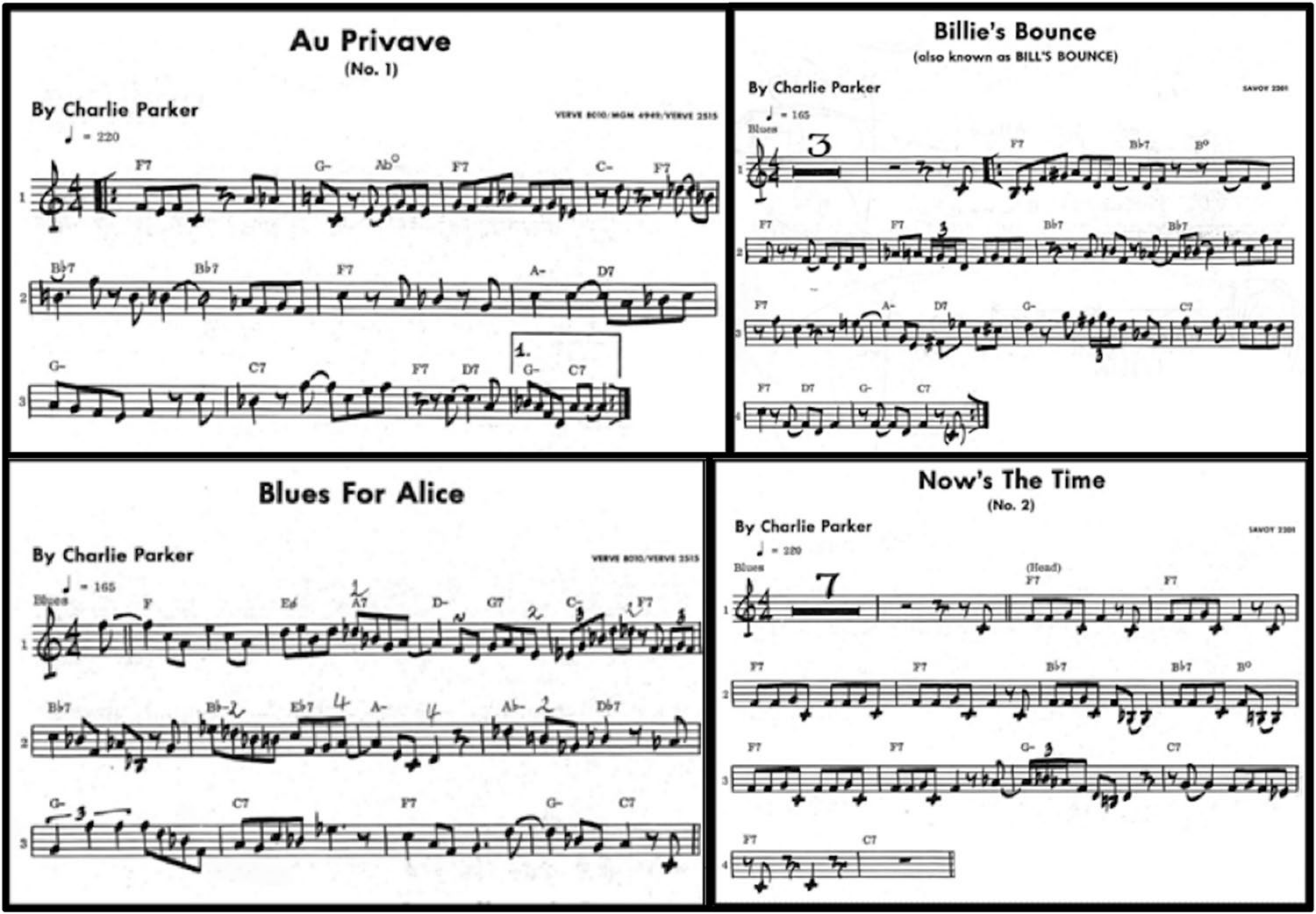
Participants learned one of these melodies at least 24 h before the scan.

This study included four important conditions affecting music performance: vocalize (Voc), imagine (Ima), improvise (Imp) and perform prelearned music (Pre). Participants familiarized themselves with four tasks designed to investigate the four mentioned conditions: vocalize prelearned (VocPre), vocalize improvised (VocImp), imagine prelearned (ImaPre), and imagine improvised (ImaImp) before the fMRI scan. During the prelearned condition, participants were prompted to vocalize or imagine one of the four scores that can be seen in Fig. 1: Au Privave, Now’s the Time, Blues for Alice, and Billies Bounce. These four melodies were chosen from the Bebop era of jazz, as the complexity of these melodies is comparable with expected improvisations^34^. Compositions are based on a standard 12-bar blues chordal progression. All melodies were memorized and rehearsed before the day of the experiment. In addition, participants were asked to complete each task module before the scan to ensure they could perform accurately within appropriate time durations. During the scan, each performance was self-paced according to a tempo specified by a metronome beat audible at the beginning of each trial resulting in a length of approximately 32 s.

Participants were instructed to imagine singing without any overt vocalization during the imagine condition and to sing (vocalize) during the vocalization condition. The VocPre and ImaPre tasks provided optimal condition control because they do not require creativity to recall from memory. Results from the two prelearned tasks (VocPre and ImaPre) were contrasted with the two improvised tasks (VocImp and ImaImp), during which participants vocalized or imagined a spontaneously improvised melody over the blues chord progression. We did not require participants to vocalize melodies and improvisations at the quality of a trained jazz singer. Here, we simply asked the musicians to vocalize as they would during a practice session (non wind instrumentalists) or during casual practice without the instrument. Such practice is common among jazz musicians and jazz students are typically asked to vocalize improvisation^34^.

### Static functional network connectivity of task paradigm

**Figure 2:**
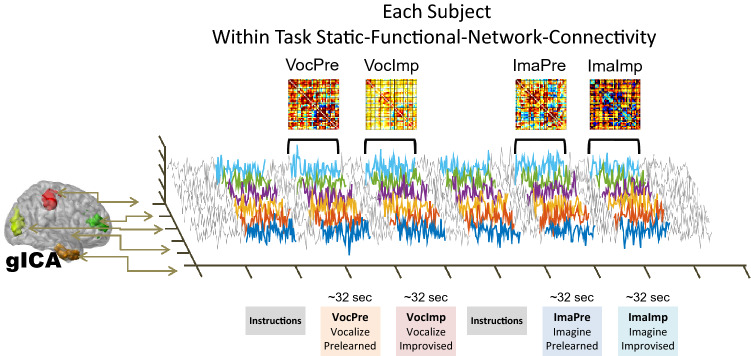
Task based static FNC procedure. Each subject went through four tasks on each session. The four tasks are: Vocalize Prelearned music (VocPre), Vocalize Improvised music (VocImp), Imagine performing Prelearned music (ImaPre), and Imagine performing Improvised music (ImaImp). Each task lasted an approximate of 32 s. Static FNC matrices were estimated using Pearson correlation coefficients applied for the duration of each task.

Data was acquired continuously through the four tasks (VocPre, VocImp, ImaPre, and ImaImp). Each task lasted an approximate of 32 s. Time courses for each network obtained from gICA thus included time lapses for all four tasks. The beginning and ending of each task were located within the time courses of each network and the time lapses were separated. Frame-wise displacement values^39^ were also calculated for each time lapse as an estimate of the amount of movement within each task. A static FNC matrix was estimated using Fisher transformed Pearson correlation coefficients of all data within each task-separated time lapse. Figure 2 illustrates the procedure that was implemented in house using Matlab (version R2019a; https://www.mathworks.com/). Finally, FNC values were orthogonalized with respect to the mean frame-wise displacement of each subject during each task. All image result plots were created in Matlab and imported into Inkscape (version 1.0; https://inkscape.org/) for illustration refinement.

### Analysis of functional network connectivity

The gICA algorithm decomposes brain signals into networks of weighted brain spatial content (spatial maps) and temporal evolution (time-courses). As explained in Fig. 2, 741 network-to-network (N2N) connectivity values were estimated using Fisher transformed Pearson correlations (z-values) from time-courses for each task and subject. A One-Way ANOVA was performed for each correlation based on the four tasks. Significance was corrected using the false discovery rate (FDR) method.

Post-hoc group tests were performed for each N2N correlation and four different contrasts including ImaPre-ImaImp, VocPre-VocImp, VocImp-ImaImp, and VocPre-ImaPre. We included two additional contrasts. A Pre-Imp = (VocPre + ImaPre) − (VocImp + ImaImp) contrast which consisted of differences between preleaned (Pre; merging VocPre and ImaPre) and improvisation (Imp; merging VocImp and ImaImp). A Voc-Ima = (VocPre + VocImp) − (ImaPre + ImaImp) contrasts consisted of differences between vocalization (Voc; merging VocPre and VocImp) and imagine (Ima; merging ImaPre and ImaImp). These analyses allowed us to identify brain networks associated with the imagination and vocalization of improvised and prelearned melodic sequences. FDR for multiple comparison correction was applied.

### Graph metric analysis

Differences in N2N connectivity suggest connectivity reconfigurations that vary for each task. Graph metrics were estimated from FNC to study these reconfigurations and their differences on each task. The four whole brain metrics tested are modularity^40^, small-world^41^, characteristic path length (char-path) and transitivity^42^. These metrics were estimated using the Brain Connectivity Toolbox (https://sites.google.com/site/bctnet/). A one way ANOVA metric was applied to each graph metric to determine task related differences.

In the context of this paper, modularity refers to a N2N connectivity pattern where networks can be grouped in strongly connected non-overlapping modules^43^. The brain connectome is organized in this modular pattern^44^. The specific modules (groups of connected networks) found in FNC are more related to particular N2N connectivity recruited during each task (see Fig. 2) than anatomical location. A related concept is functional segregation or the ability to process information within tightly connected groups of networks which is estimated using the transitivity metric. The estimation is based on the rationale that if network A is connected to network B and network B is connected to network C, then there is a high probability that A and C are also connected thus forming an network triplet of information processing^42^. The number of connected triplets is an estimation of FNC segregation. For a single network, segregation can be measured using the clustering coefficient (CC) which is a normalized assessment of the number of triplets around the network^41^. For the whole brain (all networks), transitivity is used instead of the CC which is a normalized assessment of all network triplets^42^. Another important concept is integration or the ease with which distant brain networks communicate with each other. In many instances N2N communication is mediated along a connectivity path of linked networks. The char-path metric is a whole brain integration estimate consisting on the average distance between every two endpoint networks along a path of linked networks where the distance is related to the potential for integration^45^. Information of path lengths in individual networks was estimated using eccentricity as the maximum distance between the network and any other network^46^. The human connectome is characterized by simultaneous high segregation and high integration features which are the basis of a small-world organization topology^47^. However, small-world topologies also depend on the number of connected networks and the overall distribution of this connectivity. In practice, small-world assessments compare segregation and integration against a randomly connected graph of similar characteristics (null-model) based on number of connections and their distribution^45^. The small-world assessment was performed using the tool box in https://github.com/mdhumphries/SmallWorldNess^48^ which is based on the Brain Connectivity Toolbox. We investigated if changes in both segregation and integration lead to significant small-world differences on the different tasks. Detailed mathematical and semantic descriptions of these measures can be found in^45^.

## Results

**Figure 3:**
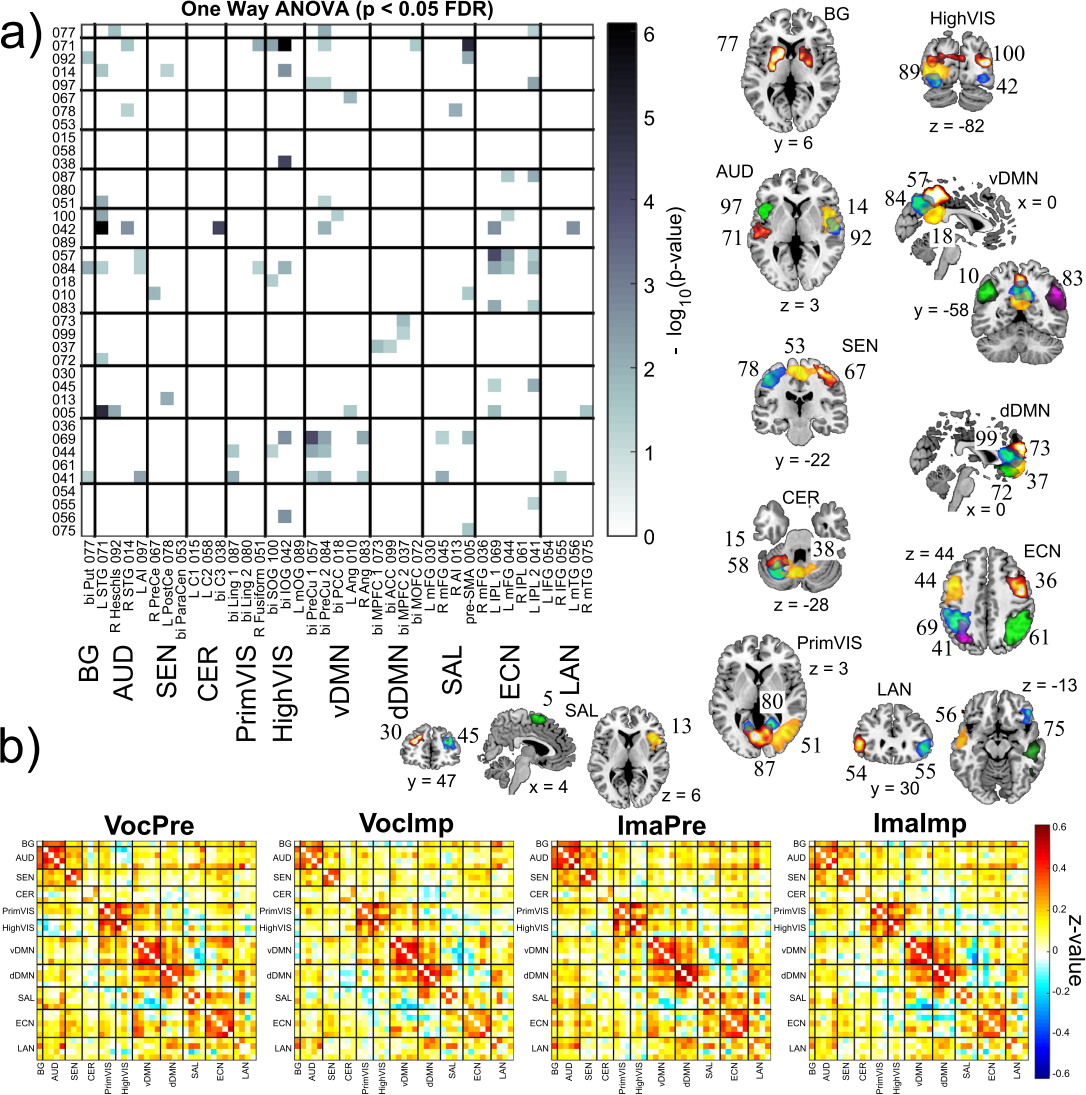
One-Way ANOVA test results considering static FNC of the four tasks: VocPre, VocImp, ImpPre and ImaImp. In (**a**), tests not passing the significance threshold p > 0.05 after FDR correction are represented in white. In (**b**), average FNC matrices for each task are presented. The figure includes spatial maps for each brain region, indicated by indexes for ease of illustration. Spatial maps were plotted using MRICRON (2MAY2016; https://www.nitrc.org/projects/mricron/). The indexes correspond to the gICA algorithm.

Figure 3 shows a composite of resulting mean correlations, spatial maps from gICA components and significant ANOVA results. Figure 3a displays a matrix of transformed p-values (transformation -log_10_(p-value) is used to improve visualization of FDR p-values). Here, significant results can be observed in almost all functional domains. The 39 spatial maps are also displayed in Fig. 3a (identified by numeric indexes; peak activations are provided in Supplementary Table). The spatial map set includes brain areas that have been previously identified as relevant for the neuroscience of creativity and music^18,24,49^: medial prefrontal cortex (MPFC; IC 73 and 37); anterior cingulate cortex (ACC; IC 99); motor areas, including left dorsal premotor (L dPM; IC 78), right dorsal premotor (R dPM; IC 67), supplementary motor area (SMA; IC 53), and pre-SMA (IC 5); cerebellum (ICs 15, 58 & 38); dorsolateral prefrontal (dlPFC) including right dlPFC (IC 36) and left dlPFC (IC 44); putamen in the limbic area (IC 77); insula (ICs 13 & 97). Notably, several components in the dorsal and ventral default mode network were also uncovered. However, Fig. 3a shows that effects are not circumscribed to a few areas, but distributed through the brain.

Figure 3b displays four average correlation matrices corresponding to each task tested in the One-Way ANOVA. Since the data is not resting state there is no expectation of a generalized anticorrelation with default mode areas. However, some task matrices in Fig. 3b show this anticorrelation pattern with higher or lower averages. The stronger correlations can be seen within each domain, except for the cerebellum and language domains, indicating that brain function is segregated on specialized functional domains.

### Comparing functional connectivity per task

Specific functional changes were investigated by examining connectivity differences between tasks based on the four contrasts ImaPre-ImaImp, VocPre-VocImp, VocImp-ImaImp and VocPre-ImaPre. In this case FDR was applied to the 741 correlations of each group difference test. However, since four tests are performed for each correlation, an additional correction was imposed by correcting FDR at p-values < 0.0125 (0.05/4).

**Figure 4:**
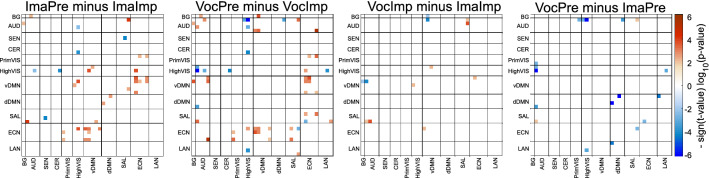
Statistical analysis of task differences. This figure displays only the significant FDR corrected p-values from the One-Way ANOVA performed for all four tasks. In addition, non-significant tests are plotted blank.

The four contrasts exhibit many significant differences as illustrated in Fig. 4. The two contrasts ImaPre-ImaImp and VocPre-VocImp show a large number of significant results with similar differences involving higher connectivity of ECN against vDMN and PrimVIS. HighVIS shows combinations of stronger (ECN) and weaker (AUD and CER) correlations with other domains. The last two contrasts VocImp-ImaImp and VocPre-ImaPre exhibit few significant results suggesting little difference whether musicians vocalize or imagine musical performance. VocImp shows weaker connectivity of vDMN against BG and AUD, but stronger connectivity of vDMN against ECN and HighVIS compared to ImaImp. VocPre exhibits weaker connectivity than ImaPre among several connections AUD-visual, AUD-dDMN, LAN-HighVIS, and within dDMN.

### Whole brain graph metrics

**Figure 5:**
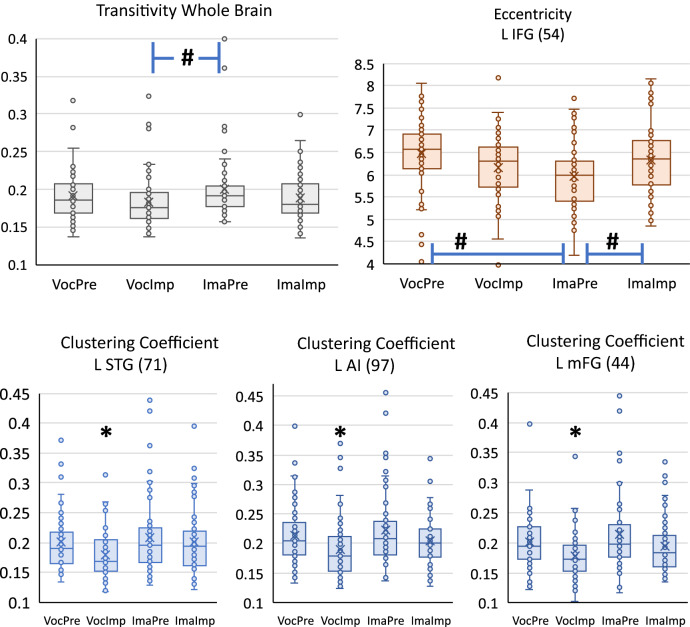
Significant group differences of graph metrics for each condition. Significant differences marked by an asterisk * indicate the lowest value among conditions. Significant differences marked as # indicate pair-wise comparisons over its corresponding line. Colors are meaningless and are used only to help differentiate among results.

Results Fig. 4 show evidence of widespread network-to-network (N2N) connectivity differences across the whole brain. These N2N differences suggest connectivity reconfigurations that vary for each task resulting in significant graph metric differences. For each graph metric, we tested the influence of frame-wise displacement by applying a MANCOVAN model (MANCOVAN is included in the GIFT software https://trendscenter.org/software/gift/). All frame-wise displacement tests were non-significant (p > 0.1) and we excluded this covariate following a reduced model approach allowing for the direct use of one way ANOVA^50^. One way ANOVA results confirmed some task differences in graph metrics after testing for normality using the Lilliefors test^51^ and applying appropriate normalization transformation (Tx): small-worldness (TX: x (no transformation), F = 3.5, p < 0.016), modularity (Tx: x^2^, F = 0.7, p = 0.55), characteristic path length (TX: x^2^, F = 7.5, p < 9e-5) and transitivity (TX: 1/x, F = 5.9, p < 7e-4). Post-hoc tests were applied to each significant metric looking to specific differences among conditions. Figure 5 shows the significant task related differences. Only Transitivity had significant differences while all other whole brain tests were excluded from Fig. 5 because their ANOVA p-values were not significant. The ImaPre task resulted in higher transitivity (higher potential for segregation) compared to VocImp. Since this contrast is not shown in Fig. 4 we provide a Supplementary Figure (ImaPre − VocImp) to show that ImaPre FNC is also generally stronger than in VocImp. Stronger correlations imply higher similarity or functional closeness among network triplets. This contrast is consisted with previously reported lesser functional connectivity during musical improvisation^33^.

### Node specific graph measures

After looking at whole brain results, we analyzed specific connectivity effects related to specific networks as illustrated in Fig. 5. A One-Way ANOVA was performed for CC and eccentricity metrics of each network. Eccentricity was significantly lower in ImaPre compared to VocPre and ImaImp in the left inferior frontal gyrus (L IFG) indicating a reduced potential for integration of this network. CC was significantly the lowest in VocImp compared to any other condition in three networks: L STG, L AI, and L mFG. These networks thus show decreased segregation in VocImp suggesting diminishing communication with other connected networks.

### Vocalize versus imagine conditions

**Figure 6:**
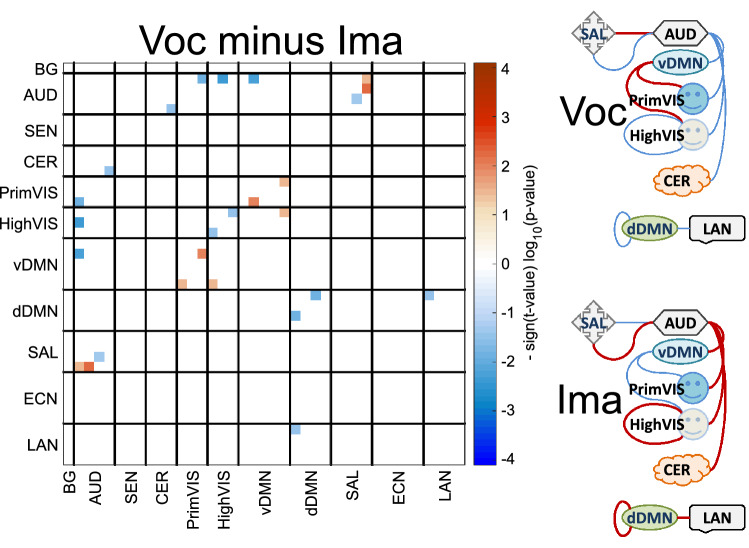
Significant results for the “vocalize minus imagine” contrast. Non-significant results are displayed in white. On the right side panel, red thick lines indicate stronger connectivity and blue thinner lines indicate weaker.

Figure 4 show patterns of strong and weak connectivity contrasts related to imaging and vocalizing. Figure 6 shows results for the contrast Voc − Ima = (VocPre + VocImp) − (ImaPre + ImaImp) with significant differences for individual connectivity values. Effects on graph metrics were not found as they probably faded after joining sample groups. A diagram of the stronger connectivity contrasts is presented on the right for ease of interpretation. The DMN shows a dual behavior depending on its dorsal (dDMN) or ventral (vDMN) components. For the Voc condition, the dDMN shows weaker connectivity with itself and with a LAN network compared to the Ima condition. However, the vDMN shows stronger connectivity with visual networks, but weaker connectivity to auditory networks in the Voc condition. The AUD domain has stronger connectivity overall in the Ima condition. There was no effect found in the ECN or SEN domains indicating that.

### Prelearned versus improvised conditions

**Figure 7:**
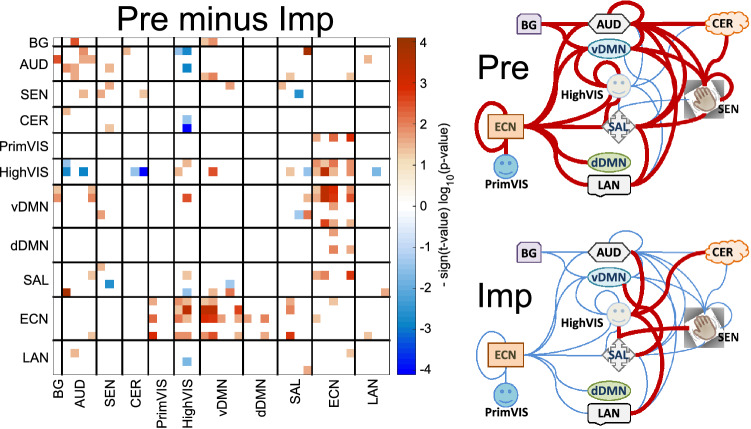
Significant results for the “prelearned minus improvised” contrast. In general, the prelearned condition exhibits significantly higher connectivity values. Non-significant results are displayed in white. On the right side panel, red thick lines indicate stronger connectivity and blue thinner lines indicate weaker.

Figure 7 summarizes the observations as ECN and AUD domains show stronger connectivity to other functional domains of the brain for the Pre condition. On the other hand, ECN is less connected during improvisation, but AUD establishes a stronger connection with areas in the HighVIS domain.

## Discussion

This work studied how the musician’s brain reconfigures its connectivity depending on the degree of creativity required during jazz music performance. We estimated static functional network connectivity from fMRI scans when scanned jazz musicians were vocalizing, imagining, improvising, or performing prelearned memorized scores. Data was analyzed with an unsupervised data-driven method to separate statistically independent brain networks and estimate their functional connectivity. A previous study conducted a similar analysis but focused on a small set of regions of interest^33^. The current analysis spans the whole brain, allowing us to observe widespread and richer effects on brain connectivity than our previous report. To our knowledge, this is the first analysis of whole brain connectivity during vocalized and imagined real-time production of creative output. Specifically, previous brain imaging studies with jazz musicians included only pianists^2,28^. The current paradigm allowed us to compare connectivity among vocally improvising musicians, providing a way to control for specific motor effects otherwise affected by the physical constraints of musical instrument manipulation.

The most important outcome in the Voc-Ima contrast is the absence of functional connectivity difference observed in ECN and SEN domains illustrated in Fig. 6. This observation suggests that connectivity of these brain networks during musical execution^19,24,49,52^ is indistinguishable whether musicians internalize or externalize their performances. Regularly, the ECN processes sensorial and internal information to coordinate behavior^16^. However, there is evidence that ECN is also elicited to accomplish mental planning, lacking of evident external behavior, which involves the imagined evaluation of a finite set of steps towards achieving a goal^53^. We can interpret these results to pinpoint one of the important purposes of ECN recruitment as aiming for the proficient planning and processing of musical related information. The difference between vocalized and imagined conditions was more evident when looking at the increased functional connectivity within the dDMN for the Ima condition, see Fig. 6. This observation is consistent with a previous study reporting increased DMN activation and connectivity during imagined tasks compared to a press button task^53^. In the current musical experiment, only specific dDMN areas were activated during the imagine condition located within the frontal pole which have been identified to process self-referential information and incorporate personally relevant stimuli as ‘mine’, i.e. self association of objects^54^. Imagined music performances could have been influenced by a more self-referenced personal experience that does not require external expression.

Connectivity differences between prelearned and improvised conditions may align with a previously suggested state of hypofrontality^18,21^. According to this view, frontal control areas are attenuated to allow bottom-up processes to drive behavior during improvisation. Figure 7 shows how our results align with a weaker connectivity of prefrontal areas (assigned to the ECN domain) during improvisation and stronger connectivity for the prelearned condition. In traditional creativity tasks (e.g., divergent thinking), ideas may be generated in an initial step by an unsupervised bottom-up process involving the DMN. In a second step, these ideas are then presumably evaluated according to applicability to the current task by a top-down control network driven by the ECN^6^. However, through training, creative experts may be able to maximize applicable ideas in the initial bottom-up stage, leading to a state in which evaluation by ECN is less necessary and thus attenuated^17,55^. This state may be particularly important during musical improvisation, where the generation occurs in real-time.

On the other hand, it is well known that performance of prelearned material is constantly evaluated to see if the actual output aligns with the encoded memory^3,56^. Here, we identified increased connectivity in the prelearned condition between ECN and DMN areas indicating higher cognitive control (see Fig. 7). In addition, ECN is more connected with visual areas, possibly because prelearned material was presented visually using traditional musical notation and the comparison between actual auditory output and the prelearned model may have involved this visual representation^57^. Finally, increased bilateral connectivity between auditory areas may involve increased evaluation processes during prelearned driven by higher level auditory cortices.

A notable effect is the reduced segregation linked to temporal, insula and frontal areas that seems to separate VocImp from the other conditions illustrated in Fig. 5. At one end Fig. 6 illustrates that connectivity within the DMN might be suppressed during the vocalize condition, avoiding interference with external goal-directed behavior seeking a proficient vocal performance^27^. At the other end in Fig. 7, improvisation reduced ECN connectivity with other networks. Notable is the reduced ECN – DMN connectivity for a free flow of musical novelty by allowing freer functioning of self-referential-default-mode areas of the brain^21^. The joint influence of both vocalizing and improvising points to a reduced number of connections which can explain the decrement in segregation observed in the VocImp against other tasks. In our previous analysis of this dataset, we found increased node activation using a traditional contrasts but less functional connectivity among six predefined areas during improvisation compared to prelearned performance^33^. Compared to VocImp, there is a trend of overall increased connectivity for the ImaPre task (see the Supplementary Figure provided). This hyperconnectivity in ImaPre is the result of combining two trends of stronger overall connectivity conditions. The prelearned had a large number of higher connectivity values than improvised (Fig. 7) and the imagine condition follow a similar overall increment of connectivity compared to vocalize. This trend played a role in the significantly lower eccentricity found in ImaPre as displayed in Fig. 5.

The pattern in which connectivity between ECN and DMN is attenuated may reflect a state of decreased top-down evaluation suggesting this state may be optimal during improvisation^18,58^. This state has been compared to the flow state in which the subject is completely immersed in an inherently motivating activity and evaluation processes are attenuated^23^. This state is dependent upon expertise in the task specific area allowing for continuous successful achievement^59^. As the flow state can be applied to many different activities, a domain general neural signature of this cognitive state has proved elusive. Yet, it has been suggested that the cognitive underpinnings of flow represent an optimal state for a specific activity^60^. Though no subjective measures of flow were collected from the current participants, several aspects of the task align with the potential for eliciting flow which is commonly reported by skilled improvisers^61^. All the participants were expert jazz improvisers. Even though none were vocalists, participants acknowledge they could perform the vocalization task without difficulty. Even though voice quality is not comparable to the standard of a trained scat vocalist, the instrumentalists could vocalize the prelearned melodies so that pitch and rhythm are easily identifiable. Expert jazz instrumentalists indeed often practice scat singing^34^. Furthermore, the harmonic context over which the improvisation was performed, the 12-measusure blues progression, is a very common tonal constraint that participants were familiar with. Yet, the task was likely immersive as the participants typically implied the harmonic structure in their single line improvisations (as is common practice in tonal jazz improvisation) without hearing an actual accompaniment. This is a typical goal of vocal practice for jazz instrumentalists and may therefore be highly motivating. Experts engaged in related motivating and immersive behaviors are very likely to experience flow^60^.

The major inherent limitation of the current paradigm compared to previous similar investigations^2^ concerns the lack of ability to analyze the improvised output. Due to technical errors, only audio recordings of 13 of the participants were captured. However, only trials in which the overall length of the improvisations aligned with the tempo given at the beginning of each trial were included in the analysis. Future research using the current vocalization task should capture high quality audio allowing for analysis of output. Furthermore, we did not ask participants to rate vividness after auditory imagine trials. This rating plus ratings of perceived flow should be included in future studies. Another important direction for future acquisition techniques will be to measure the effect of in-scanner motion during vocalization looking for ways to reduce movement variance. Gender bias toward male musicians was also a limitation. The unfortunate gender gap among expert jazz musicians is well documented^62^. Instances of female expert jazz instrumentalists to participate did not occur within the recruitment time frame. Another limitation of the cohort is the fact that instrumentalists were not expert vocal improvisers which might play a role in improvisation proficiency. However, the unusual use of vocalization for improvisation can be seen as an extra layer of creativity as musical pattern generation is less constrained by physical instrument limitations.

Outcomes in this study can be summarized as follows. First, executing prelearned music elicits higher functional connectivity than improvisation. Second, here we show for the first time that this attenuation of executive control during improvisation appears to be independent of modality (performed or imagined). Future research could adapt the imagining contrast to other domains in which actual performance of more or less creative activities is not possible within the confound of the MRI scanner (e.g. dance). Functional disconnection during improvisation has been linked to a state of mind that allows more freedom of information to flow with minimal cognitive control^21^. We found reduced integration during the VocImp task. Our results align with previous research that also found neural signatures implying improvisation is guided by bottom-up processes with less cognitive control. Importantly, our vocalization paradigm allowed for the inclusion of expert jazz musicians with many different instrument specializations implying the results may be generalizable to all jazz musicians. Future research could adapt our verbalization and imagination paradigm to other domains in which creation occurs under real-time constraints potentially identifying domain general neural signatures.

## Supplementary Information

Supplementary Information 1.

Supplementary Information 2.
