# Supplementary figures and images for: Functional network connectivity during Jazz improvisation
Source: Sci Rep. 2021 Sep 24;11:19036. doi: 10.1038/s41598-021-98332-x (PMC8463554; doi:10.1038/s41598-021-98332-x)

### FNC differences for the ImaPre – VocImp contrast

t-test lmaPre minus VocImp p<0.050

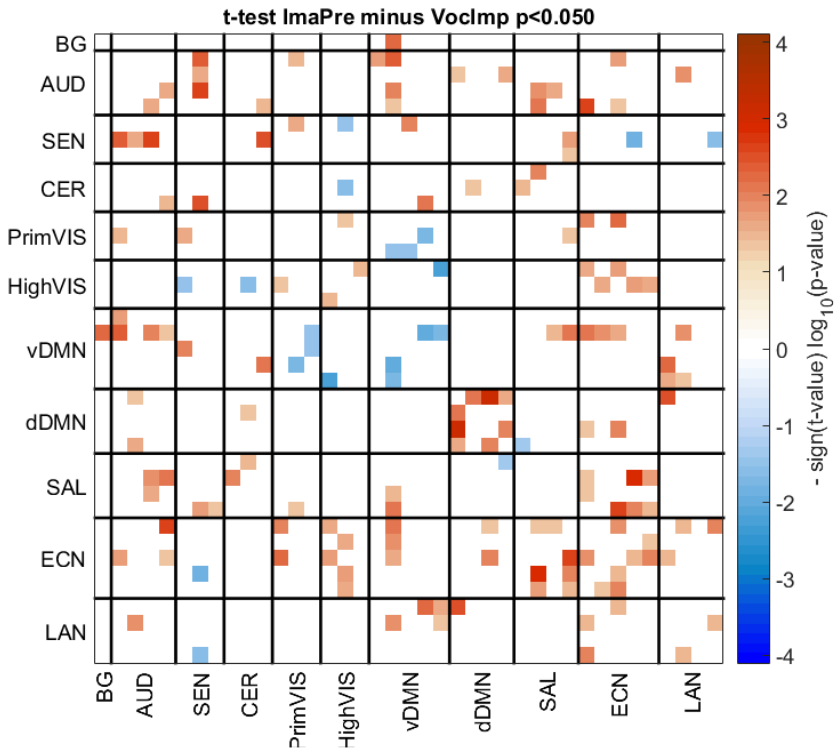

Supplement: Supplementary file 1 — Supplementary Information 1. [file 41598_2021_98332_MOESM1_ESM.pdf]
