# Supplementary material for: Functional network connectivity during Jazz improvisation
Source: Sci Rep. 2021 Sep 24;11:19036. doi: 10.1038/s41598-021-98332-x (PMC8463554; doi:10.1038/s41598-021-98332-x)
Supplement: Supplementary file 2 — Supplementary Information 2. [file 41598_2021_98332_MOESM2_ESM.docx]

Supplementary Table I. Labels and peak activations (MNI coordinates) for the 39 ICA spatial maps

| **Domain/Index** | **Color** | **Label** | **Location** | **x** | **y** | **z** |
| --- | --- | --- | --- | --- | --- | --- |
| **BG** |  |  |  |  |  |  |
| **77** | **Hot** | bi Put | Bilaterial Putamen | 18 | 21 | -4 |
| **AUD** |  |  |  |  |  |  |
| **71** | **Hot** | L STG | Left Superior Temporal | -44 | -16 | -6 |
| **92** | **Winter** | R Heschls | Right Heschls | 40 | -27 | 14 |
| **14** | **Warm** | R STG | Right Superior Temporal | 55 | -4 | -3 |
| **97** | **Green** | L AI | Left Anterior Insula/Temporal | -42 | 17 | -1 |
| **SEN** |  |  |  |  |  |  |
| **67** | **Hot** | R PreCe | Right Precentral | 30 | -26 | 66 |
| **78** | **Winter** | L PostCe | Left Precentral | -48 | -29 | 55 |
| **53** | **Warm** | bi ParaCen | Bilateral Paracentral | 2 | -26 | 60 |
| **CER** |  |  |  |  |  |  |
| **15** | **Hot** | L C1 | Left Cerebellum # 1 | -32 | -56 | -41 |
| **58** | **Winter** | L C2 | Left Cerebellum # 2 | -26 | -71 | -37 |
| **38** | **Warm** | bi C3 | Bilateral Cerebellum #3 | 12 | -59 | -27 |
| **PrimVIS** |  |  |  |  |  |  |
| **87** | **Hot** | bi Ling 1 | Bilateral Lingual 1 | -2 | -76 | 0 |
| **80** | **Winter** | bi Ling 2 | Bilateral Lingual 2 | 16 | -53 | -5 |
| **51** | **Warm** | R Fusiform | Right Fusiform | 28 | -78 | -2 |
| **HighVIS** |  |  |  |  |  |  |
| **100** | **Hot** | bi SOG | Bilateral Superior Occipital | 28 | -98 | 17 |
| **42** | **Winter** | bi IOG | Bilateral Inferior Occipital | -20 | -96 | -10 |
| **89** | **Warm** | L mOG | Left Middle Occipital | -20 | -95 | -3 |
| **vDMN** |  |  |  |  |  |  |
| **57** | **Hot** | bi PreCu 1 | Bilateral Precuneus #1 | 0 | -50 | 52 |
| **84** | **Winter** | bi PreCu 2 | Bilateral Precuneus #2 | 0 | -74 | 44 |
| **18** | **Warm** | bi PCC | Bilateral Posterior Cingulate | 0 | -50 | 15 |
| **10** | **Green** | L Ang | Left Angular | -44 | -61 | 34 |
| **83** | **Violet** | R Ang | Right Angular | 48 | -72 | 31 |
| **dDMN** |  |  |  |  |  |  |
| **73** | **Hot** | bi MPFC | Bilateral Medial Prefrontal | 0 | 59 | 27 |
| **99** | **Winter** | bi ACC | Bilateral Anterior Cingulate | 0 | 45 | 13 |
| **37** | **Warm** | bi MPFC | Bilateral Medial Prefrontal | 0 | 58 | 0 |
| **72** | **Green** | bi MOFC | Bilateral Medial Orbitofrontal | 0 | 52 | -9 |
| **SAL** |  |  |  |  |  |  |
| **30** | **Hot** | L mFG | Left Middle Frontal | -26 | 46 | 26 |
| **45** | **Winter** | R mFG | Right Middle Frontal | -50 | 14 | 32 |
| **13** | **Warm** | R AI | Right Anterior Insula | -44 | -61 | 34 |
| **5** | **Green** | pre-SMA | pre SMA | -2 | -1 | 64 |
| **ECN** |  |  |  |  |  |  |
| **36** | **Hot** | R mFG | Right Middle Frontal | 46 | 22 | 34 |
| **69** | **Winter** | L IPL 1 | Left Inferior Parietal Lobule 1 | -30 | -47 | 41 |
| **44** | **Warm** | L mFG | Left Middle Frontal | -50 | 14 | 32 |
| **61** | **Green** | R IPL | Right Inferior Parietal Lobule | 30 | -62 | 47 |
| **41** | **Violet** | L IPL 2 | Left Inferior Parietal Lobule 2 | -36 | -54 | 54 |
| **LAN** |  |  |  |  |  |  |
| **54** | **Hot** | L IFG | Left Inferior Frontal | -44 | 41 | 4 |
| **55** | **Winter** | R IFG | Right Inferior Frontal | 48 | 39 | -2 |
| **56** | **Warm** | L mTG | Left Middle Temporal | -55 | -7 | -19 |
| **75** | **Green** | R mTG | Right Middle Temporal | 59 | -27 | -6 |
